# Urbanization and Global Health: The Role of Air Pollution

**Published:** 2018-11

**Authors:** Qing WANG

**Affiliations:** 1. Dept. of Economic, School of Business, Dalian University of Technology, Panjin, Liaoning, China; 2. Dept. of Social Medicine and Maternal & Child Health, School of Public Health, Shandong University, Jinan, Shandong, China

**Keywords:** Urbanization, Global health, Air pollution

## Abstract

**Background:**

The world is experiencing the biggest wave of urban growth in history. The association between urbanization and health at the global level, as well as the role of air pollution, has not been studied. We aimed to examine the effect of urbanization on global health and the role of air pollution.

**Methods:**

Unbalanced panel data comprising 3, 093 observations of 163 countries for 1990–2012 from the World Bank database was used. An infinite distributed lag model was applied to estimate the contemporary and long-term effects of urbanization on health outcomes measured by mortality, under-five mortality, infant mortality, life expectancy at birth(all; female; and male).

**Results:**

Urbanization was positively related to global health in the short term and long term. In the short run, 1% increase in urbanization was associated with reduced mortality, under-five mortality, and infant mortality of 0.05%, 0.04%, and 0.04%, respectively, as well as increased life expectancy of 0.01 year. The effects of urbanization were stronger for high-income countries. However, air pollution undermined the positive impacts of urbanization on health.

**Conclusion:**

Although urbanization leads to improved global health, air pollution undermines the positive effects of urbanization on health. Developing sustainable urbanization practices is crucial for addressing the challenges of pollution caused by urbanization.

## Introduction

Urbanization refers to the process of expansion in the proportion of population residing in urban areas. The world is experiencing the biggest wave of urban growth in history. Currently, over 50% of the global population lives in urban areas- this is 3.9 billion, and by 2030, this number will rise to about 5 billion (1). Urbanization leads to great social and economic progress. Although urbanization is related to a series of human welfare outcomes, its influence on population health is not so clear. Urban residents may benefit from improved sanitation, infrastructure, and access to health services; however, they may be confronted with other issues, including unhealthy lifestyles and environmental pollution in urban areas (2–5).

The early empirical studies on the relationship between urbanization and health focus on developed or high-income countries, which reveal that the association of urbanization and health is complex. The majority of studies found that urbanization results in health improvement (6–9). However, in urban areas, where the bulk of the population excessively concentrates, unsatisfying sanitation and health promotion intervention leave much to be desired; thus, urbanization may be related to poorer health (10–12). Urban growth is expected to occur faster in low-income or developing countries. The urbanization in some countries is related mostly to economic development. In developing countries, the largest cities are concentrated in the largest economies (e.g. Brazil in Latin America and China in Asia). “There are also some cities in other regions (e.g., sub-Saharan Africa) where movement occurs despite economic stagnation and in others whose urban population is increased by the movement thereof people displaced by wars, civil strife, or drought” (5). Thus, the empirical literature on the association between urbanization and health laid emphasis on either large cities or ghettos in developing or low-income countries. Few studies found urbanization improves health, most of them asserted that urbanization results in a health penalty (13–20). It is unknown whether, and if so, to what extent, urbanization affects global health.

The heterogeneous findings across countries may lie on the facts that the levels of pollution in low-income or developing countries are often several orders of magnitude higher than those in high-income or developed countries. Most of that pollution is coming from urbanization development (21). Air pollution causes obvious health damage to humans (22). Lives are cut short by heart disease, stroke, lung disease or cancer that are triggered or exacerbated by air pollutants (23, 24). Air pollution is expected to play a more important role in the association of urbanization and health outcomes, not studied in the literature (25). Our study attempts to fill these gaps by investigating two questions: 1) How does urbanization affect health in the world with various levels of urbanization? 2) What is the role of air pollution?

We evaluated the relationship between urbanization and health in 163 countries for which the relevant data for analysis are available from the World Bank. Our findings not only supported the improved health outcomes related to urbanization but also indicated that air pollution might threaten the positive health influence of urbanization.

## Materials and Methods

To estimate the effects of urbanization on mortality, unbalanced panel data comprising 3093 observations from 163 countries for the period 1990–2012. The primary data source used in this study is World Development Indicators compiled by the World Bank (26).

In this study, mortality rate, under-five mortality, infant mortality rate (per 1,000 live births), and life expectancy (all, female, male) were applied to demonstrate global health status. Mortality rate and life expectancy at birth are both major indicators determined for evaluation in most epidemiological studies and clinical trials. In addition, mortality rate and life expectancy are “hard” data with easily understandable meaning of the health status outcome (27).

An infinite distributed lag model (IDL) was applied to estimate the contemporary and lagged effect of urbanization on health. In the model, no lag length needs to be taken into account for the lagged effect of urbanization on health, therefore the model solves the problem of choosing the correct lag length (28). The IDL model, on the basis of Neumayor’s study, can be presented as follows with panel data context (28, 29):

[1] $$Y_{\mathrm{it}}=a+bY_{\mathrm{it}-1}+cX_{\mathrm{it}}+dU_{\mathrm{it}}+fP_{\mathrm{it}}+gU_{\mathrm{it}}\times P_{\mathrm{it}}+hP_{\mathrm{it}}^{2}+e_{\mathrm{it}},$$

In Equation [1], *Y_it_* represents the health outcomes for country *i* at time *t*; *U_it_* is the urbanization level of country *i* at time *t; Pit* and *P_it_^2^* are air pollution intensity and its quadratic term, respectively. Air pollution intensity refers to the indicator of total greenhouse gas emissions per Gross Domestic Product (GDP) (kt of CO2 equivalent/hundred billion dollars). *U_it_×P_it_* is the interaction of air pollution emissions intensity and urbanization.

*X_it_* is a vector representing other covariates for city *i* at time *t*. To represent the channels through which macroeconomic variables may influence health, *X_it_* includes population density; gender ratio; the proportion of population aged 65 year and older; GDP growth level; primary school enrollment rate; official development assistance per capita; and share of urban population with improved sanitation (30).

*u_i_* represents the country dummy variable, and *v_it_* is the idiosyncratic error term.

Following Arellano and Bond (1991), the Generalized Method of Moments (GMM) estimator was used to estimate the equation [1]. The basic idea of this estimator is to use all prior dependent variables that are valid instruments. Robust standard errors were employed, which are robust toward arbitrary autocorrelation as well as heteroscedasticity. The LLC (Levin–Lin–Chu) and IPS (Im–Pesaran–Shin) tests rejected the hypothesis of unit roots (*P*<0.01), the series are stationary. A Sargan test proved the validity of the model.

The contemporaneous effects of urbanization could be calculated by *d+the mean of air pollution emissions intens*ity×g, while the long-term effects could be computed by the contemporaneous effects/(1-b) for b<1; for b>1, the long-term effects denoted the health effect of urbanization in the next ten years, calculated by the contemporaneous effects× (1-b^10^)/(1-b). Analyses were also stratified by income level, which is set according to the World Bank’ criteria for 2012 (26). Statistical analyses were performed using STATA 14.0.

## Results

### Statistical description

Country characteristics are presented in Table 1. For the entire sample period, the average mortality rate per 1,000 individuals was 9.08; the average under-five mortality was 66.44% and the infant mortality was 45.73%.

**Table 1:** Characteristics of 163 countries for the period 1990–2012 by income

***Variable***	***All (N=3093)***	***High income countries/Middle and high income countries (N=956)***	***Low income countries/Middle and Low income countries (N=2137)***
	***Mean (Std. Dev)***	***Mean (Std. Dev.)***	***Mean (Std. Dev.)***
Mortality	9.08	7.03	10.02
	(3.79)	(2.57)	(3.89)
Under-five mortality	66.44	30.45	82.34
	(54.39)	(25.06)	(56.23)
Infant Mortality	45.73	24.25	55.52
	(31.20)	(16.71)	(31.50)
Life expectancy at birth	64.93	70.69	62.33
	(9.08)	(5.77)	(9.10)
Male life expectancy at birth	67.20	73.26	64.37
	(9.82)	(5.77)	(9.90)
Female life expectancy at birth	62.75	68.04	60.37
	(8.46)	(5.69)	(8.43)
Urbanization level	0.46	0.59	0.41
	(0.20)	(0.19)	(0.18)
Air pollution intensity	7.37	2.53	9.57
	(24.49)	(4.03)	(29.27)
Gender ratio	0.50	0.50	0.50
	(0.01.)	(0.02)	(0.01)
Population density	96.43	134.51	79.07
	(148.13)	(213.04)	(101.53)
The proportion of 65 yr and older	0.05	0.06	0.05
	(0.03)	(0.03)	(0.03)
GDP growth level	0.04	0.04	0.04
	(0.05)	(0.07)	(0.04)
Primary school enrollment rate	0.99	1.06	0.96
	(0.19)	(0.10)	(0.21)
Share of urban population with improved sanitation	0.71	0.88	0.63
	(0.25)	(0.11)	(0.25)
Official development assistance per capita	56.74	54.49	57.90
	(86.09)	(80.50)	(88.51)

Note: Data are presented as mean (standard deviation)

Life expectancy at birth was estimated at 64.93 year, with female life expectancy being 62.95 year, which was lower than the male life expectancy of 67.20 year.

The mean urbanization level was 46.26%. Air pollution intensity was 7.37 KT of CO2 equivalent per hundred billion dollars GDP.

Overall, health outcomes have fluctuated over countries. High-income countries fared better in health indicators, showing lower mortality and longer life expectancy (mortality: 7.03; life expectancy: 70.69) than low-income countries (mortality: 10.02; life expectancy: 62.33). Urbanization in high-income countries (59%) was significantly greater than that in low-income countries (41%). In contrast with urbanization and health, air pollution intensity in high-income countries (2.53 KT of CO2 equivalent per hundred billion dollars GDP) was significantly lower than in low-income countries (9.57 KT of CO2 equivalent per hundred billion dollars GDP).

### Multivariate regression results

The relationship between urbanization and health outcomes as well as the role of air pollution was estimated (Table 2). Urbanization brought benefits to health outcomes measured by mortality, under-five mortality, infant mortality, life expectancy, female life expectancy, and male life expectancy at birth (*P*<0.01). A 1% increase in the urbanization level led to a reduction of 0.06%, 0.05%, and 0.06% in the mortality rate, under-five mortality, and infant mortality, respectively, as well as an increase of 0.01 in the life expectancy at birth (female: 0.002; male: 0.01), without considering the effect of air pollution. However, air pollution played an adverse role in the association of urbanization and health outcomes. After adjusting for air pollution, a 1% increase in the urbanization level was related to a lower 0.05% (−1%*5.85+1%*0.11*7.37) mortality rate, a lower 0.04‰ (−1%*5.18+1%*0.10*7.37) under-five mortality, and a lower 0.04% (−1%*5.86+1%*0.11*7.37) infant mortality, respectively. A 1% increase in the urbanization level was associated with a longer 0.01 (1%*1.28–1%*0.1*7.37) life expectancy at birth, a 0.02 (1%*2.32–1%*0.10*7.37) longer female life expectancy at birth. However, for male, urbanization decreased the life expectancy at birth by 0.00 (1%*0.58–1%*0.10*7.37).

**Table 2:** Associations between urbanization level, air pollution and health outcomes

***VARIABLES***	***Mortality***	***Under-five mortality***	***Infant Mortality***	***Life expectancy at birth***	***Male life expectancy at birth***	***Female life expectancy at birth***
	***Coef. (Std. Err.)***	***Coef. (Std. Err.)***	***Coef. (Std. Err.)***	***Coef. (Std. Err.)***	***Coef. (Std. Err.)***	***Coef. (Std. Err.)***
Urbanization level	−5.85^***^	−5.18^***^	−5.86^*^	1.28^***^	0.58^***^	2.32^***^
	(1.22)	(1.19)	(3.55)	(0.14)	(0.13)	(0.17)
Air pollution intensity	−0.02^***^	−0.06^***^	−0.04^***^	0.04^***^	0.04^***^	0.04^***^
	(0.00)	(0.02)	(0.01)	(0.00)	(0.00)	(0.00)
Interaction of air pollution intensity and urbanization	0.11^***^	0.10^*^	0.11^***^	−0.10^***^	−0.10^***^	−0.10^***^
	(0.00)	(0.00)	(0.00)	(0.00)	(0.00)	(0.00)
Air pollution intensity’s quadratic	37.11	7405.02^***^	3165.93^***^	−471.51^**^	−315.62	−380.41
	(166.67)	(1483.7)	(449.8)	(209.33)	(199.52)	(250.74)
Gender ratio	6.07^***^	33.69^***^	2.34	−31.79^***^	−0.31.02^***^	−34.26^***^
	(1.09)	(10.42)	(3.18)	(1.35)	(1.29)	(1.62)
Population density	−0.00	−0.00^***^	−0.00^***^	0.01^***^	0.00^***^	0.00^***^
	(0.00)	(0.00)	(0.00)	(0.00)	(0.00)	(0.00)
The proportion of 65 yr and older	1.09	−53.4^***^	−21.63^***^	7.71^***^	4.98^***^	19.28^***^
	(0.75)	(7.97)	(2.49)	(1.03)	(0.98)	(1.210
GDP growth level	−0.41^***^	−1.64^***^	−1.77^***^	1.46^***^	1.61^***^	0.01^***^
	(0.07)	(0.60)	(0.18)	(0.08)	(0.07)	(0.00)
Primary school enrollment rate	0.75^***^	−8.20^***^	−4.20^***^	−0.86^***^	−0.61^***^	−0.10^***^
	(0.47)	(0.56)	(0.17)	(0.06)	(0.06)	(0.08)
Share of urban population with improved sanitation	1.64^***^	15.28^***^	5.65^***^	−4.70^***^	−3.51^***^	−5.57^***^
	(0.09)	(0.90)	(0.29)	(0.12)	(0.12)	(0.14)
Official development assistance per capita	−0.00^***^	−0.00^***^	−0.00	0.00^***^	0.00^***^	0.00^***^
	(0.00)	(0.00)	(0.00)	(0.00)	(0.00)	(0.00
Lag of independentvariable	0.97^***^	0.95^***^	0.95^***^	1.04^***^	1.05^***^	1.01^***^
	(0.00)	(0.00)	(0.00)	(0.00)	(0.00)	(0.00)

Note: Coefficient (Robust standard error) were calculated by the infinite distributed lag model, on the basis of Neumayor’s study

*** *P*<0.01,

** *P*<0.05,

* *P*<0.1 as determined using the infinite distributed lag model

Table 3 presents the results when the analysis stratified by income level. Urbanization improved health outcomes for both high-income countries and low-income countries without considering the impact of air pollution. However, the favorable effects of urbanization on health outcomes were significantly reduced by air pollution in low-income countries. Air pollution played a significant and adverse role in the association of urbanization and health outcomes in low-income countries judging from the interaction of air pollution and urbanization. Whereas, compared to low-income countries, the adverse role of air pollution was insignificant for the majority of health indicators in high-income countries.

**Table 3:** Associations between urbanization level and health outcomes by income level

***VARIABLES***	***Mortality***	***Under-five mortality***	***High income Infant Mortality***	***countries Life expectancy at birth***	***Male life expectancy at birth***	***Female life expectancy at birth***
	***Coef. (Std. Err.)***	***Coef. (Std. Err.)***	***Coef. (Std. Err.)***	***Coef. (Std. Err.)***	***Coef. (Std. Err.)***	***Coef. (Std. Err.)***
Urbanization level	−1.45^***^	−4.68^***^	−3.26^***^	2.70^***^	2.38^***^	2.98^***^
	(0.14)	(0.48)	(0.27)	(0.16)	(0.15)	(0.22)
Air pollution intensity	−0.01	0.12^***^	0.10^***^	−0.11^***^	−0.10^***^	−0.10
	(0.00)	(0.00)	(0.00)	(0.00)	(0.00)	(0.00)
Interaction of total greenhouse gas emissions and urbanization	0.13^**^	−0.13	0.04	0.01	0.13^***^	−0.14
	(0.02)	(0.11)	(0.10)	(0.00)	(0.02)	(0.11)
Air pollution intensity’s quadratic	−20706.18	−184914.44^***^	−95085.23^***^	125016.08	196533.81	24341.51
	(20916.91)	(61583.11)	(34234.79)	(245080.11)	(228289.04)	(33764.82)
Lag of independent variable	0.92^***^	0.92^***^	0.92^***^	0.92^***^	0.93^***^	0.92^***^
	(0.01)	(0.00)	(0.00)	(0.01)	(0.00)	(0.01)
Low-income countries						
VARIABLES	Mortality	Under-five mortality	Infant Mortality	Life expectancy at birth	Male life expectancy at birth	Female life expectancy at birth
	Coef. (Std. Err.)	Coef. (Std. Err.)	Coef. (Std. Err.)	Coef. (Std. Err.)	Coef. (Std. Err.)	Coef. (Std. Err.)
Urbanization level	−0.05	−1.19^***^	−2.35^***^	0.86^***^	1.41^***^	0.62^***^
	(0.16)	(0.19)	(0.54)	(0.19)	(0.19)	(0.20)
Air pollution intensity	−0.02^***^	−0.07^***^	−0.05^***^	0.04^***^	0.03^***^	0.04^***^
	(0.00)	(0.02)	(0.01)	(0.00)	(0.00)	(0.00)
interaction of total greenhouse gas emissions and urbanization	0.12^***^	0.14^*^	0.13^***^	−0.14^***^	−0.12^***^	−0.11^***^
	(0.02)	(0.10)	(0.02)	(0.02)	(0.01)	(0.01)
Air pollution intensity’s quadratic	134.27	8279.82^***^	3950.86^***^	−1039.14^***^	−1190.84^***^	−723.44^***^
	(146.73)	(1711.344)	(501.90)	(191.32)	(188.97)	(202.72)
Lag of independent variable	0.99^***^	0.94^***^	0.94^***^	1.05^***^	1.05^***^	1.04^***^
	(0.00)	(0.00)	(0.00)	(0.00)	(0.00)	(0.00)

Note: Control variables included urbanization; air pollution intensity and its quadratic term; the interaction of air pollution emissions intensity and urbanization; population density; gender ratio; the proportion of population aged 65 yr and older; GDP growth level; primary school enrollment rate; official development assistance per capita; share of urban population with improved sanitation and lag of independent variable. Coefficient (Robust standard error) were calculated by the infinite distributed lag model, on the basis of Neumayor’s study

*** *P*<0.01,

** *P*<0.05,

* *P*<0.1 as determined using the infinite distributed lag model

The contemporary and long-term effects of urbanization on health outcomes were favorable controlling for air pollution. And the long-term effects were bigger. In the long run, a 1% increase of urbanization was associated with reduced mortality, under-five mortality, and infant mortality of 1.68%, 0.89%, and 1.01%, respectively, as well as increased life expectancy of 0.07 yr. Furthermore, the effects of urbanization were stronger for high-income countries (Table 4).

**Table 4:** The contemporary and long-term effects of urbanization on health outcomes

***Variable***	***All***		***High-income countries***		***Low-income countries***	
	***Contemporary effects***	***Long-term effects***	***Contemporary effects***	***Long-term effects***	***Contemporary effects***	***Long-term effects***
Mortality	−5.04^a^	−167.98^b^	−1.12	−14.01^b^	——^d^	——
Under-five mortality	−4.44	−88.86^b^	−4.68	−58.50^b^	0.15	2.50^b^
Infant Mortality	−5.05	−100.99^b^	−3.26	−40.75^b^	−1.11	−18.43^b^
Life expectancy at birth	0.54	6.52^c^	2.70	33.75^b^	−0.48	−6.03^c^
Male life expectancy at birth	−0.16	−1.97^c^	2.71	38.70^b^	0.26	3.29^c^
Female life expectancy at birth	1.58	16.56^c^	2.98	37.25^b^	−0.43	−5.20^c^

Note: Control variables included urbanization; air pollution intensity and its quadratic term; the interaction of air pollution emissions intensity and urbanization; population density; gender ratio; the proportion of population aged 65 yr and older; GDP growth level; primary school enrollment rate; official development assistance per capita; share of urban population with improved sanitation and lag of independent variable.

a The contemporary effect was the coefficient of urbanization + the coefficient of the interaction of air pollution emissions intensity and urbanization*the mean of air pollution emissions intensity in the infinite distributed lag model;

b The long-term effects could be computed by the contemporary effect/(*1-* the coefficient of the lag of independent variable in the infinite distributed lag model);

c the long-term effects were calculated by a ten years’ effect, which is the contemporaneous effects*(1-b10)/(1-b) (b: the coefficient of the lag of independent variable in the infinite distributed lag model);

d Since the coefficient of urbanization on mortality in the low-income countries were not significant, effects of urbanization on mortality were not documented

## Discussion

According to the World Bank, the ratio of urban population at the global level has already exceeded 53.86% in 2015, and this will continue to rise in the coming decades (31). Urbanization has been considered as one of the most important development strategies. Urbanization works as the engine of socio-economic development (32). At the same time, urbanization puts pressure on urban ecosystems, which may cause health damage (33). There is a significant variation in urbanization along with air pollution across the regions. Some countries are able to develop a satisfying urbanization with low air pollution in year 1990–2012 (e.g., Denmark (urbanization: 83.09%; air pollution intensity: 1.01 kt of CO2 equivalent/hundred billion dollars); some others achieve high-level urbanization, but air pollution is also serious (e.g., Russia (urbanization: 68.93%; air pollution intensity: 5.912 kt of CO2 equivalent/hundred billion dollars)); others’ urbanization maintain a low-level, but air pollution has been severe (e.g., Vietnam (urbanization: 21.71%; air pollution intensity: 5.66 kt of CO2 equivalent/hundred billion dollars)).

Such a variation provides data sets to estimate the association between urbanization and health, as well as the role of air pollution. No study has examined the effects of urbanization along with air pollution on health from a global perspective. Therefore, the aim of this study was to assess the health performance of urbanization at a global level.

Overall, urbanization led to improved global health. However, air pollution undermined the favorable impacts of urbanization on health and resulted in a health penalty. The health penalty is stronger for the low-income countries, where air pollution intensity was more serious. Some countries even find a substantial health penalty of urbanization that reverses the health effects linked to urbanization (e.g., China) (19).

Urbanization is undeveloped in low-income countries and air pollution is anticipated to keep rising along with urbanization for a long time (1, 21). From a policy perspective, how to balance urbanization and air pollution to achieve sustainable development deserves attention. This study may still provide a new method for establishing urbanization policies as well as policies related to both individual health and public health concerns. First, developing sustainable urbanization practices is crucial for addressing the challenges of pollution caused by urbanization (34, 35). Governments should take health and environmental protection into consideration for urbanization construction and evaluation. In this regard, governments and various non-governmental organizations worldwide have been increasingly introducing low-carbon measures to guide the practices of sustainable urbanization toward better health, including the UN’s Millennium Declaration (36), the Istanbul Declaration of the North Atlantic Treaty Organization (37) the Mexico City Government, Green Plan (38), and the Government of Singapore’s Green Plan (39). The promotion of sustainable and low-carbon urbanization in the last decade has resulted in many positive experiences, and sharing and learning the best practice between different countries can significantly contribute to the global mission of sustainable urbanization (40).

Second, our findings provide justification for interventions of health that target exposure to air pollution along with the improved urbanization level. Economic development cannot solve all the health problem, what is worse, economic development may bring health penalty (e.g. air pollution brought by urbanization). In this case, we cannot rely on economic development to tackle down all the health issues. As such, basing policy on urbanization and economic development may be insufficient to protect population health. More precise measures should be determined and implemented to meet the health needs of those people. For instance, public health policy should provide information to them on how to deal with the adverse effects of air pollution. Further, health outcomes among individuals living in low-income countries were particularly susceptible to fluctuations in air quality. The difference of health status between high-income countries and low-income countries are expected to be widened if air pollution continues to be serious in the latter countries. Special attention should be paid to population health in low-income countries. The governments and international organizations should work to enhance urbanization as well as focus prevention strategies and environmental regulation in the low-income countries.

This study extends the research on the subject of health effects of urbanization by testing the relationship between the two all over the world and the role of air pollution. However, the limitations of this study must be taken into consideration.

While we highlight air pollution as an important mechanism, other channels exist through which urbanization may impact health. Unhealthy lifestyles and other pollution forms are significant determinants. There is a growing concern over the pollution of water and solids and lifestyles linked to urbanization (41, 17, 19). Social cohesion, mutual trust, and social integration are also related to global health (42). Future research must consider these points when analyzing the effects of urbanization on population health.

## Conclusion

Using an unbalanced panel data of 163 countries for 1990–2012 from the World Bank database, this study evaluated the short-run and long-term effects of urbanization on global health. The role of air pollution was also estimated. Overall, global health gained benefits from urbanization. However, air pollution may undermine the health improvement from urbanization. Government should seek balance between urbanization and air pollution regulation to achieve sustainable development.

## Ethical considerations

Ethical issues (Including plagiarism, informed consent, misconduct, data fabrication and/or falsification, double publication and/or submission, redundancy, etc.) have been completely observed by the authors.
